# Author Correction: Perfluoroalkyl substance pollutants activate the innate immune system through the AIM2 inflammasome

**DOI:** 10.1038/s41467-022-33408-4

**Published:** 2022-09-27

**Authors:** Li-Qiu Wang, Tao Liu, Shuai Yang, Lin Sun, Zhi-Yao Zhao, Li-Yue Li, Yuan-Chu She, Yan-Yan Zheng, Xiao-Yan Ye, Qing Bao, Guang-Hui Dong, Chun-Wei Li, Jun Cui

**Affiliations:** 1 MOE Key Laboratory of Gene Function and Regulation, State Key Laboratory of Biocontrol, School of Life Sciences, Sun Yat-sen University, Guangzhou, Guangdong China; 2 Department of Otolaryngology, The First Affiliated Hospital, Sun Yat-sen University, Guangzhou, Guangdong China; 3 Guangdong Provincial Engineering Technology Research Center of Environmental Pollution and Health Risk Assessment, Department of Occupational and Environmental Health, School of Public Health, Sun Yat-sen University, Guangzhou, Guangdong China

**Correction to**: *Nature Communications* 10.1038/s41467-021-23201-0, published online 18 May 2021

The original version of this Article contained an error in Supplemental Figure 13b. The third image from the left in the upper row shows a lung sample of Aim2-/- mice under PFOS treatment instead of “Donor WT Recipient WT”, what is stated in the description.

While the description should stay the same, the correct figure panel should be inserted, with the WT>WT Mock /lung staining sample now replacing the incorrect sample.

The HTML has been updated to include a corrected version of the Supplementary Information.

## Updated Supplementary Information

Updated Supplementary Table S1

