# Supplementary material for: Perfluoroalkyl substance pollutants activate the innate immune system through the AIM2 inflammasome
Source: Nat Commun. 2021 May 18;12:2915. doi: 10.1038/s41467-021-23201-0 (PMC8131593; doi:10.1038/s41467-021-23201-0)
Supplement: Supplementary file 1 — Supplementary Information [file 41467_2021_23201_MOESM1_ESM.pdf]

Supplementary information

Perfluoroalkyl substances pollutants activate innate immune system through the AIM2 inflammasome

Li-Qiu Wang[1], Tao Liu[1], Shuai Yang[1], Lin Sun[1], Zhi-Yao Zhao[1], Li-Yue Li[1], Yuan-Chu She[1], Yan-Yan Zheng[1], Xiao-Yan Ye[1], Qing Bao[1], Guang-Hui Dong[2], Chun-Wei Li[1]*, Jun Cui[1]*

[1] MOE Key Laboratory of Gene Function and Regulation, State Key Laboratory of Biocontrol, School of Life Sciences, and Department of Otolaryngology, The First Affiliated Hospital, Sun Yat-sen University, Guangzhou, Guangdong, China

[2] Guangdong Provincial Engineering Technology Research Center of Environmental Pollution and Health Risk Assessment, Department of Occupational and Environmental Health, School of Public Health, Sun Yat-sen University, Guangzhou, Guangdong, China

*Correspondence should be addressed to J.C. (cuij5@mail.sysu.edu.cn) and C.L. (hi_chunwei@aliyun.com).

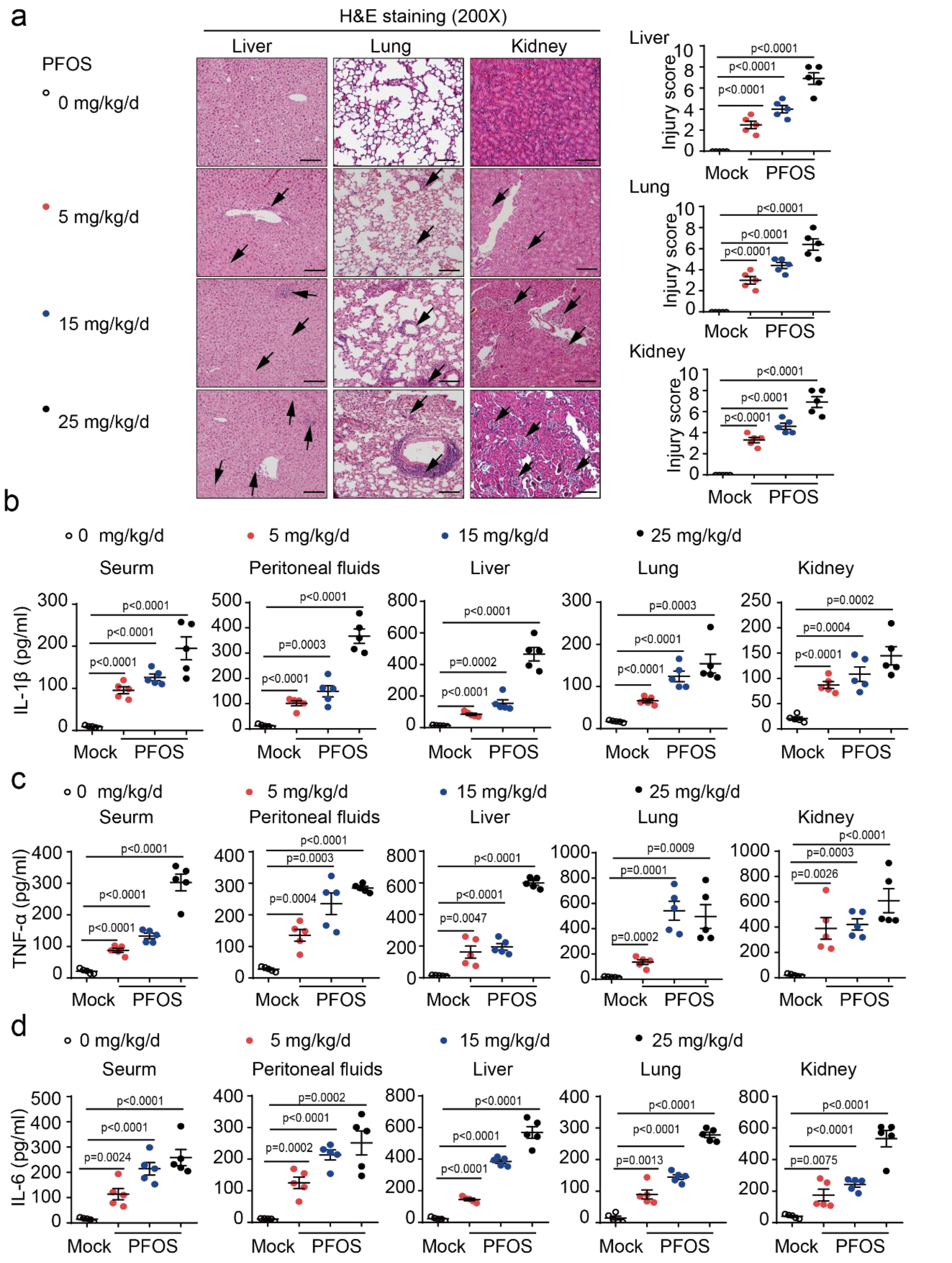

Supplementary Fig. 1 Acute exposure of PFOS induced tissue damage and the production of pro-inflammatory cytokines in mice in a dose-dependent manner. a-d C57BL/6 mice (female, 6 weeks old, n=5) were treated with PFOS (5 (red plots), 15 (blue

plots) or 25 (black plots) mg/kg body weight per day) or treated with PBS containing 2 % Tween-80 (Mock) by intraperitoneal injection for 5 days. At 5 days post-treatment, indicated tissues were stained with hematoxylin-eosin (H&E) and assayed using a light microscope with ×200 mangnification (**a**). Scale bar, 100 μm. The tissue injury scores were determined and averaged in 5 randomly selected nonoverlapping fields from respective individual mouse tissue sections. At 5 days post-treatment, indicated tissues of treated mice were isolated and cultured for 24 h, and supernatants were analyzed by ELISA for IL-1β (**b**), TNF-α (**c**) and IL-6 (**d**). In (**a-d**), all error bars, mean values ± SD, P-values were determined by unpaired two-tailed Student's t test of n=5 independent biological mice per group. Source data are provided as a Source Data file.

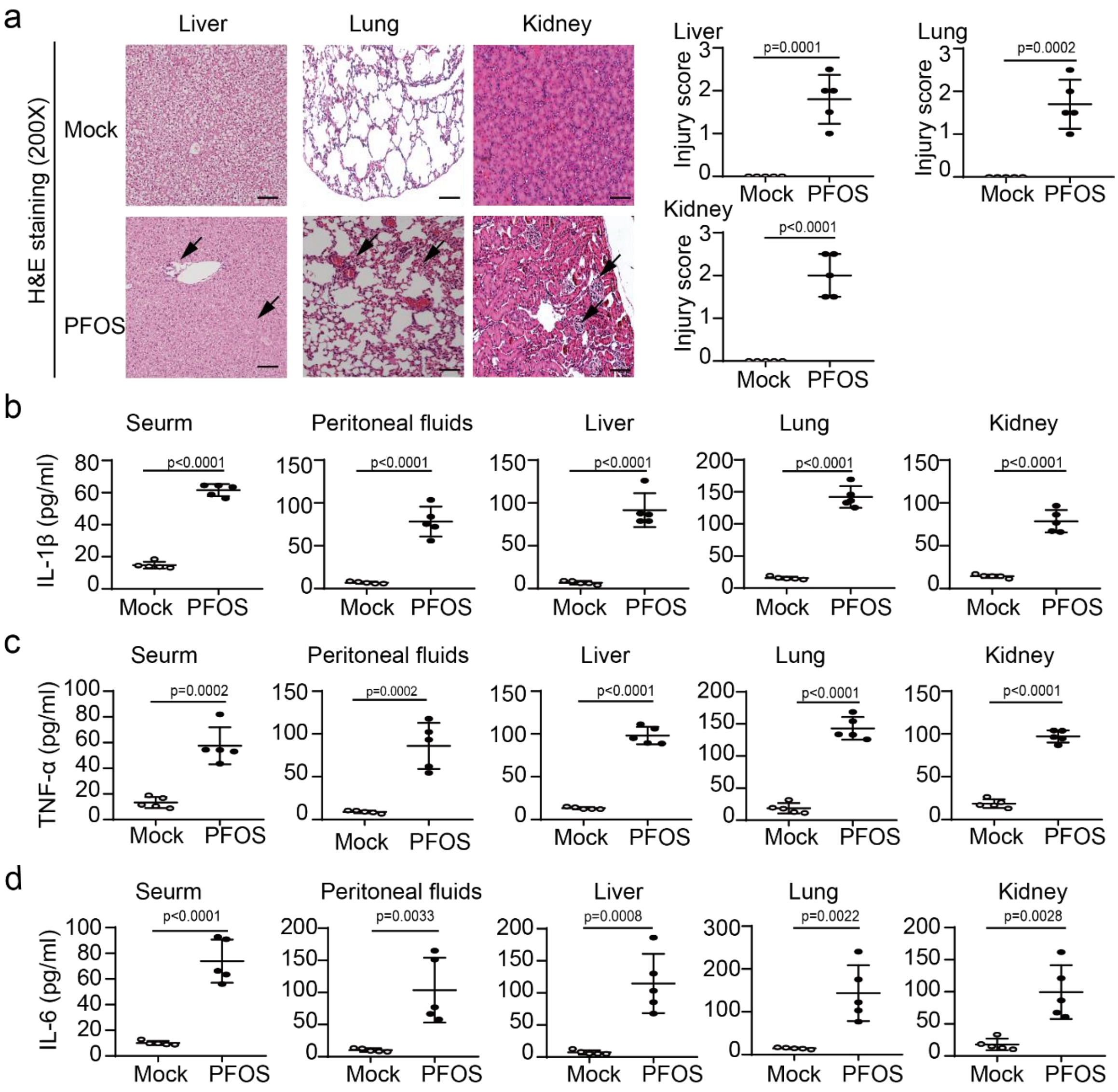

Supplementary Fig. 2 Chronic low-dose exposure of PFOS induced tissue damage and the production of pro-inflammatory cytokines in mice. a-d C57BL/6 mice (female, 6 weeks old, n=5) were treated with PFOS (0.066 mg/kg body weight per day) or treated with PBS containing 2 % Tween-80 (Mock) by intraperitoneal injection for 30 days. At 30 days post-treatment, mice liver, lung and kidney tissue were stained with hematoxylin-eosin (H&E) and assayed using a light microscope with ×200 mangnification (**a**). Scale bar, 100 μm. The tissues (liver, lung, and kidney) injury scores were determined and averaged in 5 randomly selected nonoverlapping fields from respective individual mouse tissue sections. All histology analyses were conducted in a blinded manner. At 30 days post-treatment, the liver, lung and kidney of treated mice were isolated and cultured for 24 h,

and supernatants were analyzed by ELISA for IL-1β (**b**), TNF-α (**c**) and IL-6 (**d**). In (**a-d**), all error bars, mean values ± SD, P-values were determined by unpaired two-tailed Student's t test of n=5 independent biological mice per group. Source data are provided as a Source Data file.

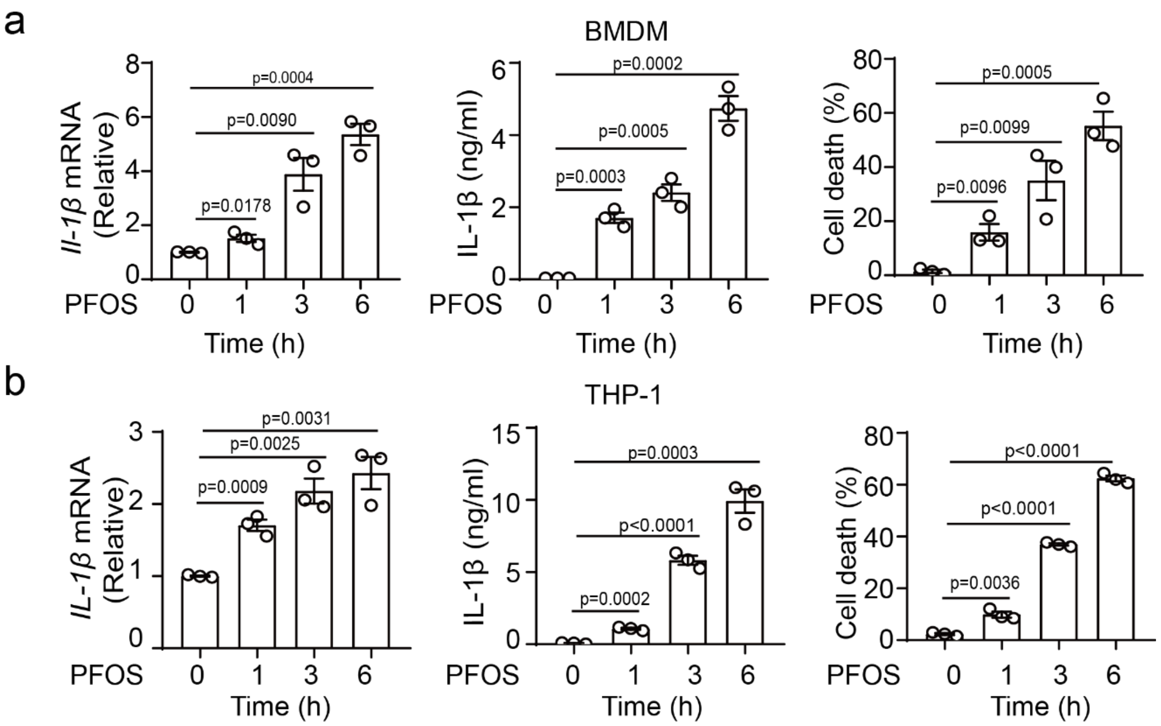

Supplementary Fig. 3 PFOS induces IL-1β expression and production, and cell death in a time-dependent manner. a-b Bone marrow-derived macrophages (BMDMs) (**a**) or THP-1-deirived macrophages (**b**) were treated with PFOS (150 μM) in indicated time points, cell lysates were collected to detect mRNA levels of *IL-1β*, and cell supernatants were collected to measure IL-1β release and cell death. In (**a-b**), all error bars, mean values ± SEM, P-values were determined by unpaired two-tailed Student's t test of n=3 independent biological experiment. Source data are provided as a Source Data file.

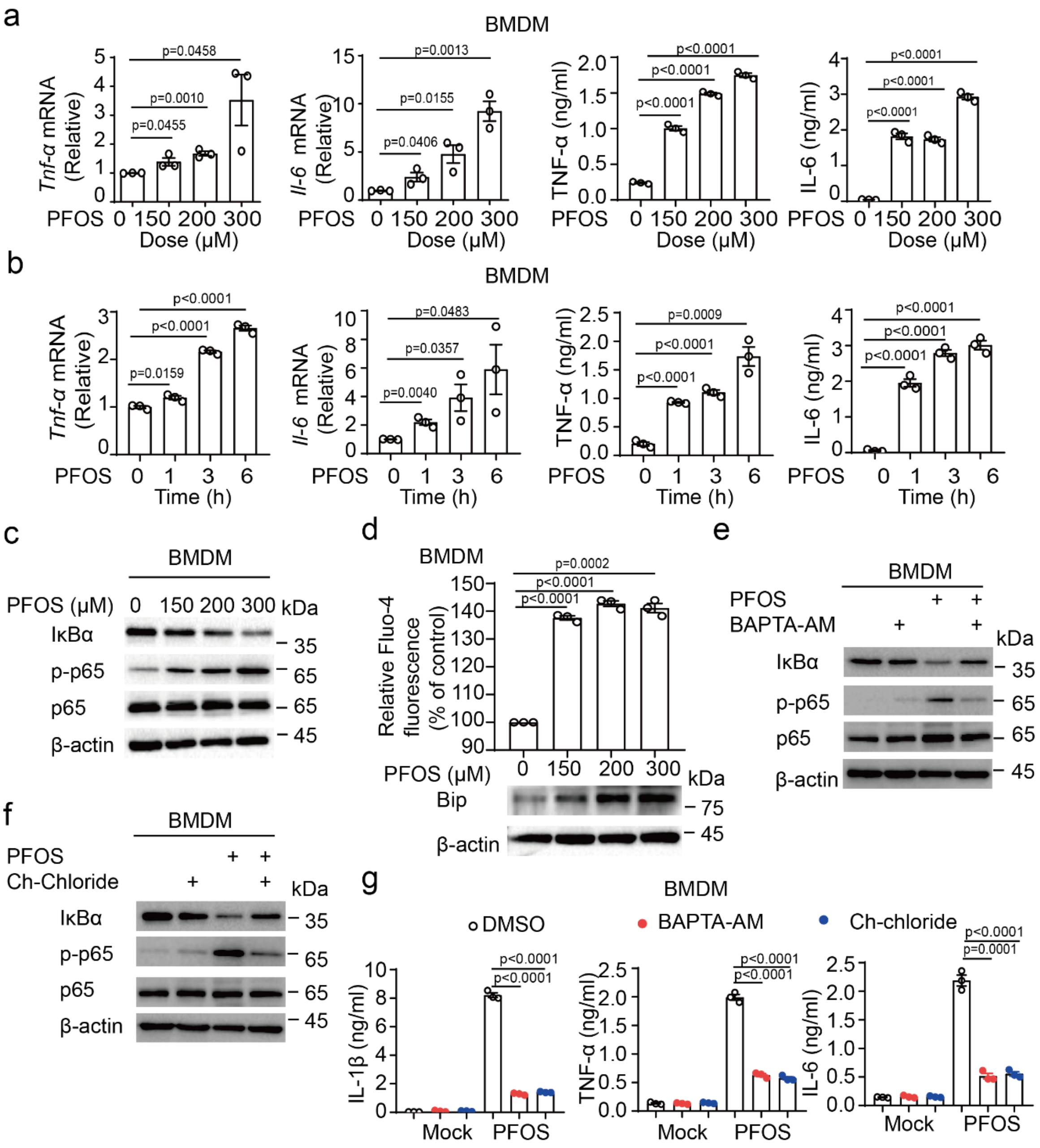

Supplementary Fig. 4 PFOS triggers Ca^{2+}-PKC-dependent NF-κB signaling pathway in BMDMs. a-b Bone marrow-derived macrophages (BMDMs) were treated with PFOS in a dose-dependent manner for 6 h (**a**) or in time-dependent manner with 150 μM PFOS (**b**). The expression and production of TNF-α and IL-6 were determined. **c-d** Bone marrow-derived macrophages (BMDMs) were treated with PFOS in a dose-dependent manner for 6 h. Immunoblot analysis of NF-κB signaling pathway was performed with indicated antibodies in BMDMs (**c**). The cytosolic Ca^{2+} ($[Ca^{2+}]c$) in BMDMs with PFOS as indicated

doses for 6 h, was assessed by measuring the fluorescence of cells stained with Fluo-4 AM via using fluorescence microplate reader (excitation and emission wavelength at 488 nm and 520 nm, respectively) (**d**). $[Ca^{2+}]c$ was presented as the percentage relative to the value in control group. **e-g** Bone marrow-derived macrophages (BMDMs) were pretreated with Ca^{2+} chelator (BAPTA-AM, 50 μM for 1 h, red plots) or PKC inhibitor chelerythrine chloride (Ch-Chloride, 10 μM for 1 h, blue plots) and subsequently treated with PFOS (150 μM, 6 h). Cell lysates were collected for immunoblot analysis. Cell supernatants were harvested to determine the release of proinflammatory cytokines (IL-1β, TNF-α and IL-6) by ELISA. In (**a**, **b**, **d** and **g**), all error bars, mean values ± SEM, P-values were determined by unpaired two-tailed Student's t test of n=3 independent biological experiment. For (**c**-**f**), similar results are obtained for three independent biological experiments. Source data are provided as a Source Data file.

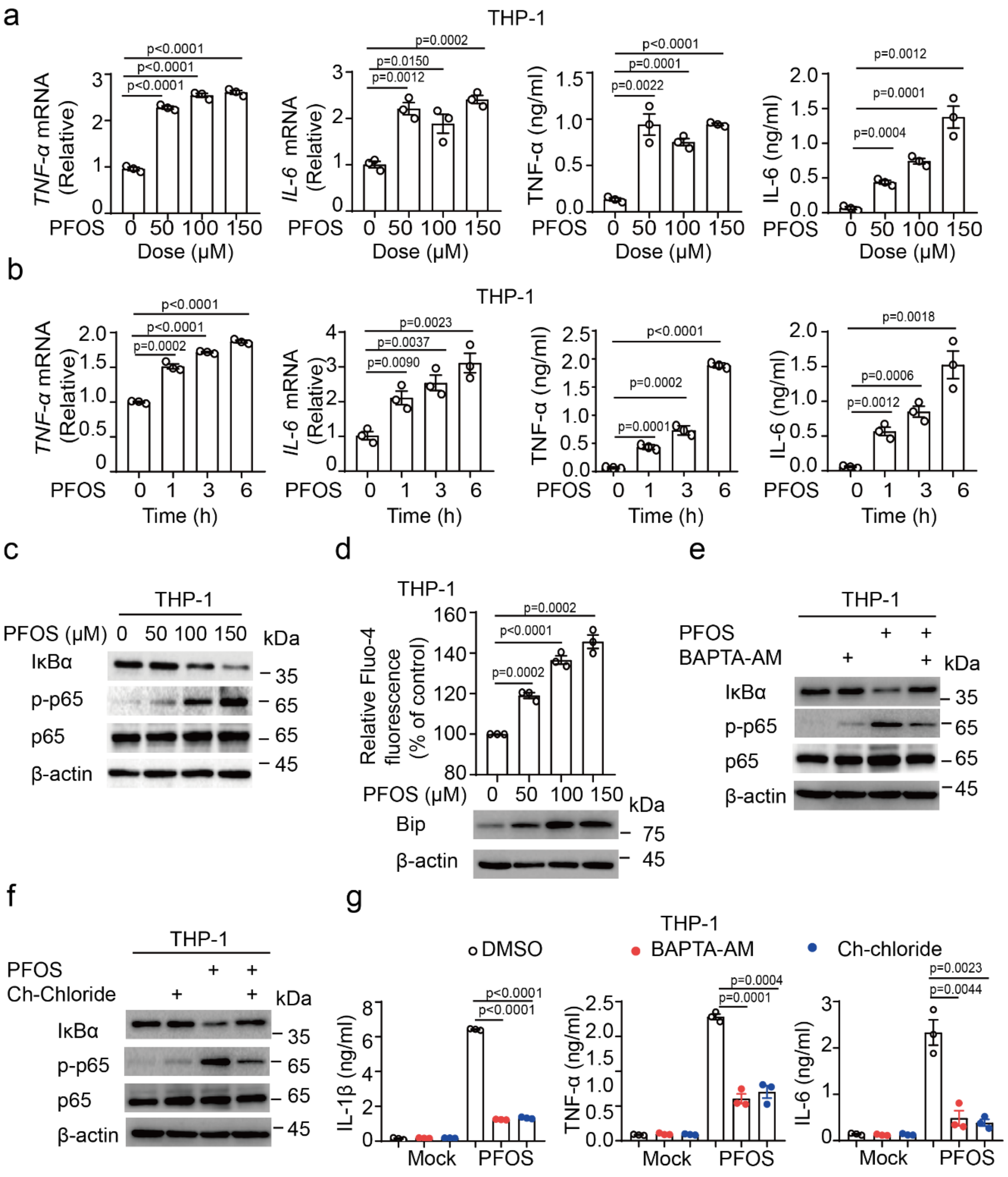

Supplementary Fig. 5 PFOS-induced NF-κB signaling activation was mediated through Ca^{2+}-PKC dependent pathway in THP-1-derived macrophages. a-b THP-1-derived macrophages were stimulated with PFOS in a dose-dependent manner for 6 h (**a**) or in time-dependent manner with 150 μM PFOS (**b**). The expression level and release of TNF-α and IL-6 were determined. **c-d** THP-1-derived macrophages were treated with PFOS in a dose-dependent manner for 6 h. Immunoblot analysis of NF-κB signaling

pathway were detected with indicated antibodies (**c**). The cytosolic Ca^{2+} ($[Ca^{2+}]c$) in THP-1-derived macrophages with PFOS as indicated doses for 6 h, was performed by measuring the fluorescence of cells stained with Fluo-4 AM via using fluorescence microplate reader (excitation and emission wavelength at 488 nm and 520 nm, respectively) (**d**). **e-g** THP-1-derived macrophages were pretreated with Ca^{2+} chelator (BAPTA-AM, 50 μM for 1 h, red plots) or PKC inhibitor chelerythrine chloride (Ch-Chloride, 10 μM for 1 h, blue plots) and then stimulated with PFOS (150 μM, 6 h). Cell lysates were harvested for immunoblot analysis. Cell supernatants were collected to determine the production of proinflammatory cytokines (IL-1β, TNF-α and IL-6) by ELISA. In (**a**, **b, d** and **g**), all error bars, mean values ± SEM, P-values were determined by unpaired two-tailed Student's t test of n=3 independent biological experiment. For (**c-f**), similar results are obtained for three for three independent biological experiments. Source data are provided as a Source Data file.

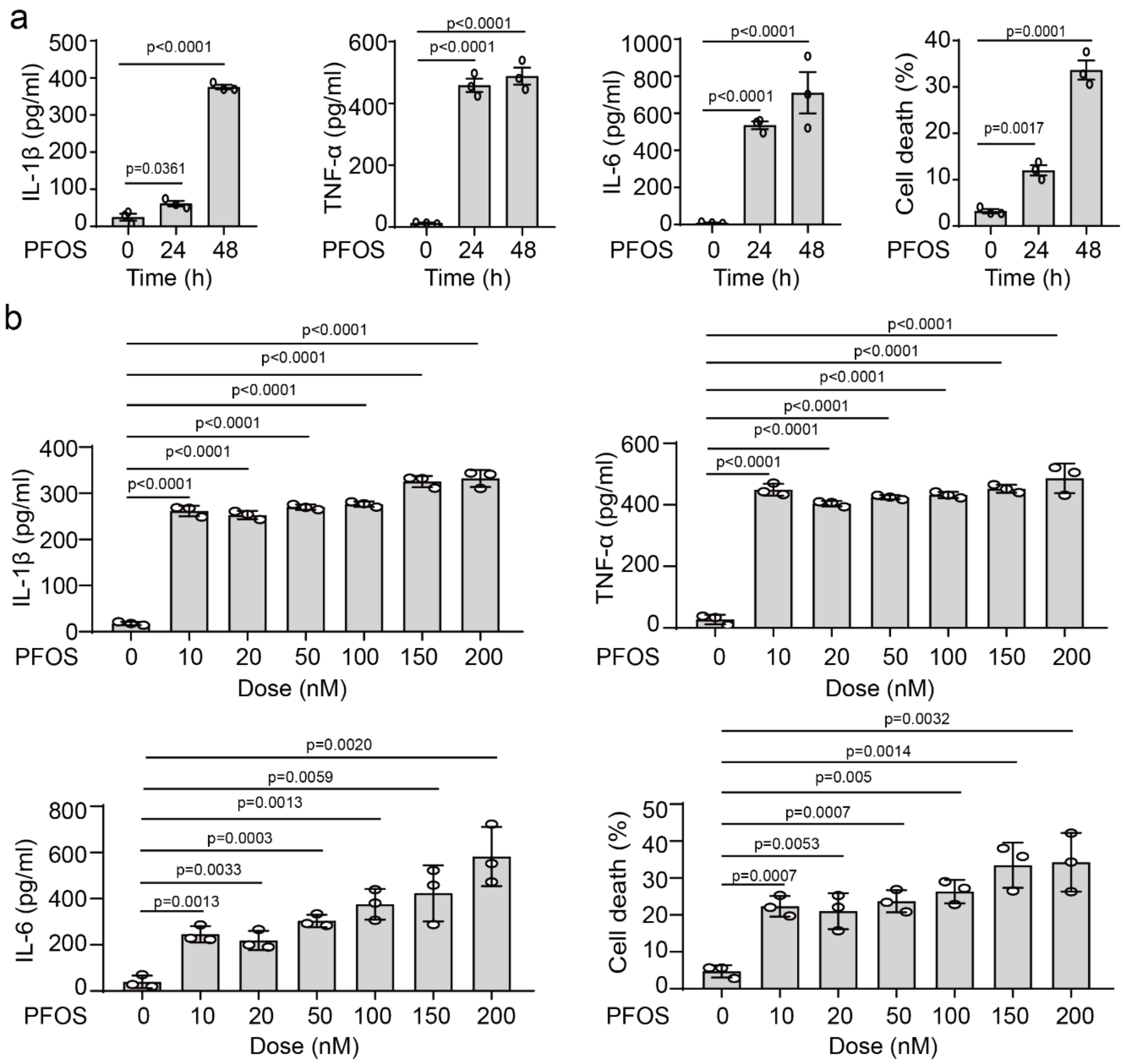

Supplementary Fig. 6 Low concentrations of PFOS triggered proinflammatory cytokines production and cell death in THP-1-derived macrophages. a-b THP-1-derived macrophages were treated with PFOS (200 nM) as indicated time points (**a**) or as indicated doses for 48 h (**b**). The production of IL-1β, TNF-α and IL-6 and the cell death were determined. All error bars, mean values ± SEM, P-values were determined by unpaired two-tailed Student's t test of n=3 independent biological experiment. Source data are provided as a Source Data file.

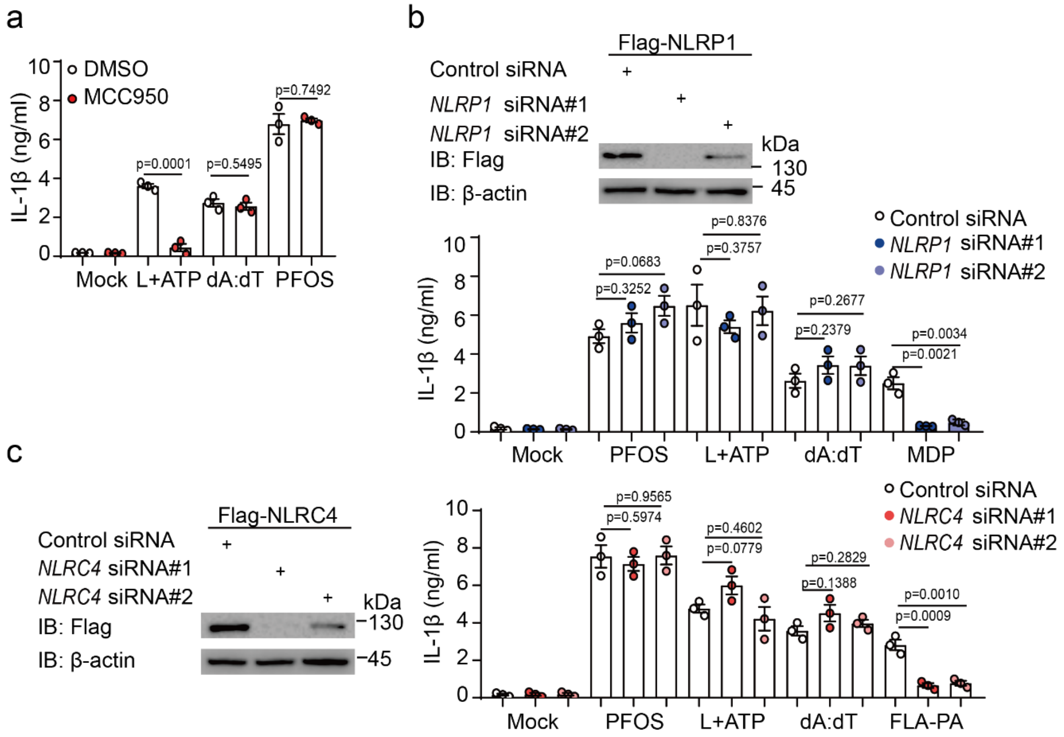

Supplementary Fig. 7 PFOS-induced IL-1β production is independent on NLRP3/NLRP1/NLRC4 inflammasome. a THP-1-derived macrophages were pretreated with NLRP3 specific inhibitor MCC950 (10 μM, red plots) for 1 h and subsequently treated with PFOS (150 μM, 6 h) or poly (da:dT) (2 μg/ml, 6 h) or pre-treated with LPS (200 ng/ml, 3 h) followed by ATP (5 mM, 6 h). Cell supernatants were collected to measure IL-1β production by ELISA. **b-c** *NLRP1* knockdown (KD) (*NLRP1* siRNA#1:deep blue plots; #2: light blue plots) (**b**) or *NLRC4* KD (**c**) (*NLRC4* siRNA#1: deep red plots; #2: light red plots) THP-1-derived macrophages were treated with PFOS (150 μM, 6 h), AIM2 agonist poly (dA:dT) (2 μg/ml, 6 h), NLRP1 agonist muramyl dipeptide (MDP, 500 ng/mL, 6 h), NLRC4 agonist FLA-PA (0.1 μg/mL, 6 h), or pre-treated with LPS (200 ng/ml, 3 h) before ATP (5 mM, 6 h). IL-1β release was measured in the supernatants by ELISA. All error bars, mean values ± SEM, P-values were determined by unpaired two-tailed Student's t test of n=3 independent biological experiment. Source data are provided as a Source Data file.

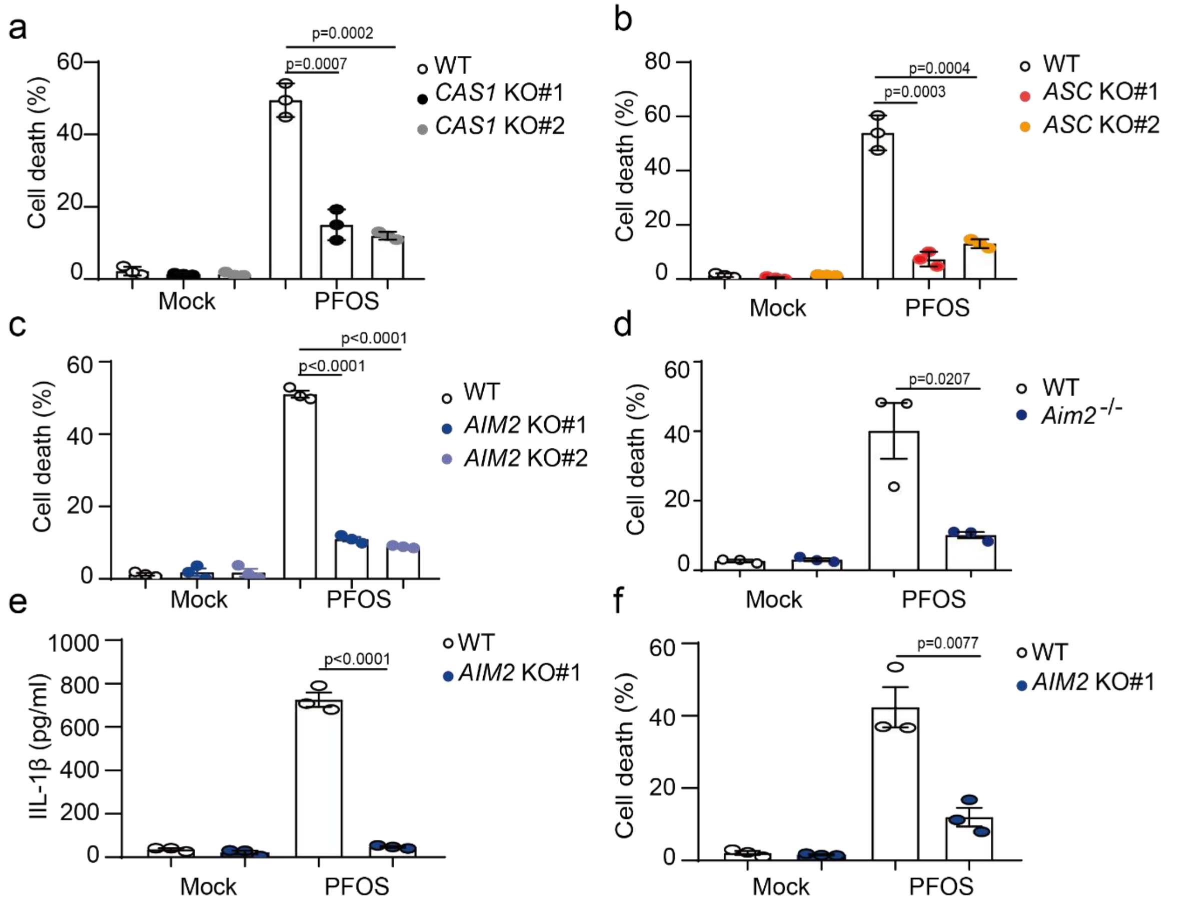

Supplementary Fig. 8 AIM2 inflammasome is required for IL-1β production and cell death induced by PFOS with different doses. a-d WT (white), *CAS1* KO (**a**, KO#1: black, KO#2: grey), *ASC* KO (**b**, KO#1: red plots; KO#2: orange plots), *AIM2* KO (**c**, KO#1: deep blue plots; KO#2: light blue plots) THP-1-derived macrophages or *Aim2*$^{-/-}$ Bone marrow-derived macrophages (BMDMs) (**d**, blue plots) were treated with PFOS (150 μM, 6 h). Cell supernatants were then collected to measure cell death by LDH release. **e-f** *AIM2* KO#1 (blue plots) THP-1-derived macrophages were treated with PFOS (200 nM, 48 h). IL-1β and LDH release were measured in the supernatants. All error bars, mean values ± SEM, P-values were determined by unpaired two-tailed Student's t test of n=3 independent biological experiment. Source data are provided as a Source Data file.

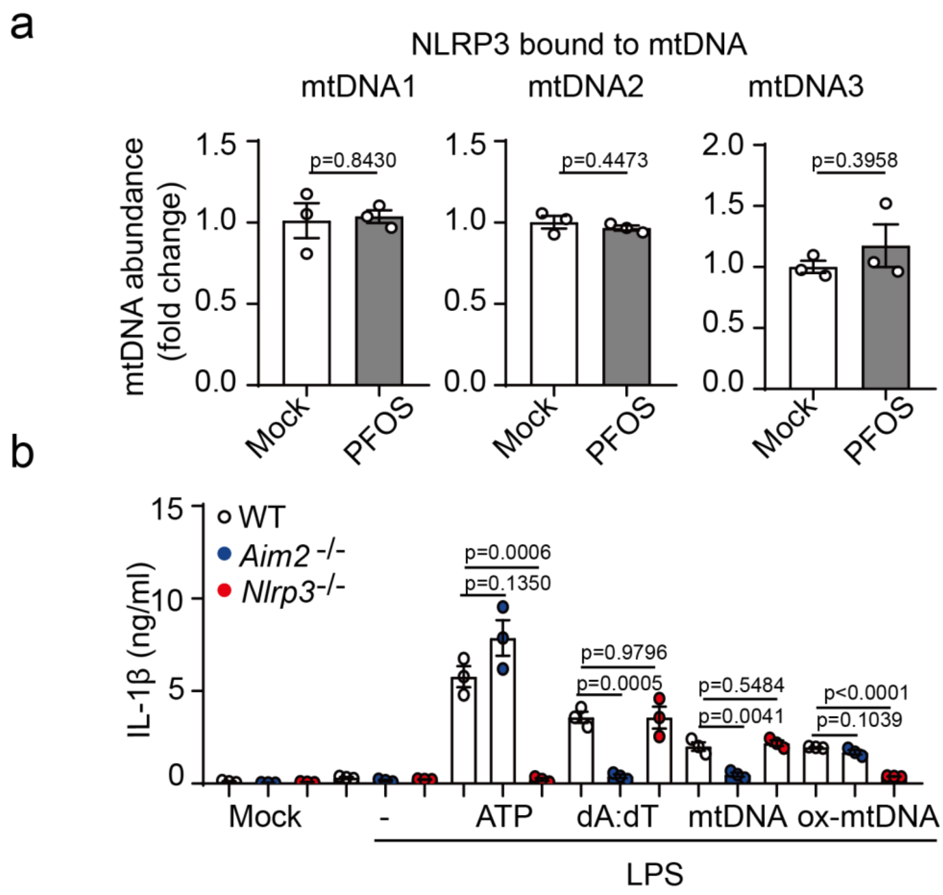

Supplementary Fig. 9 Non-oxidized mtDNA induces AIM2 inflammasome activation and oxidized-mtDNA activates NLRP3 inflammasome. a Relative enrichment of DNA in AIM2-pulldown material from Mock or PFOS (150 μM, 6 h) condition. qPCR for three sets of primers that amplify fragments from different regions of the human mtDNA. **b** WT, *Aim2*$^{-/-}$ (blue plots) and *Nlrp3*$^{-/-}$ (red plots) Bone marrow-derived macrophages (BMDMs) primed with LPS (500 ng/ml, 3 h) were incubated with NLRP3 agonist ATP (5 mM, 6 h), AIM2 agonist poly (dA:dT) (2 μg/ml, 6 h), synthetic mtDNA (non-oxidized mtDNA, 2 μg/ml, 6 h), oxidized-mtDNA (2 μg/ml, 6 h), and then the release of IL-1β was measured by ELISA. All error bars, mean values ± SEM, P-values were determined by unpaired two-tailed Student's t test of n=3 independent biological experiment. Source data are provided as a Source Data file.

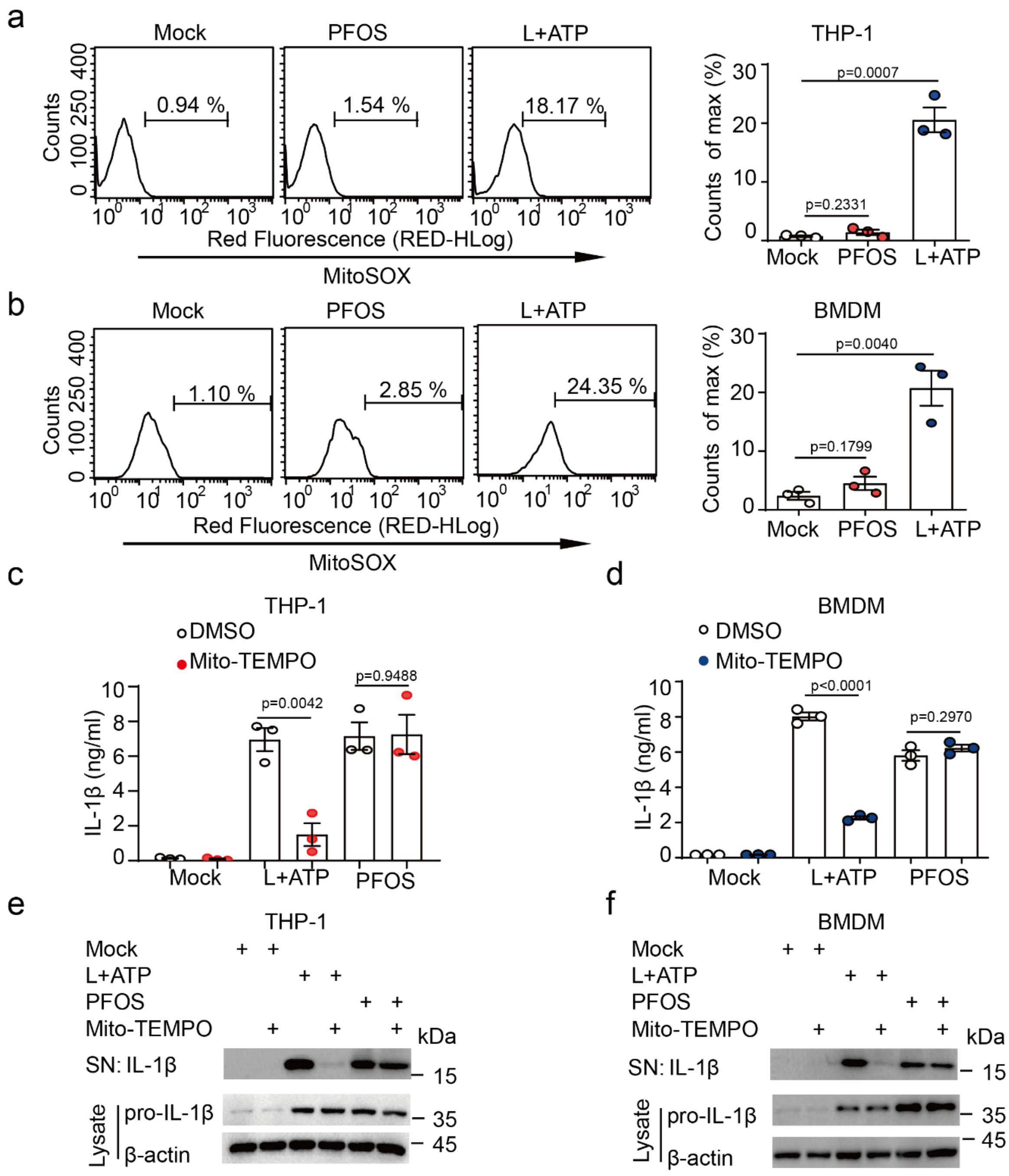

Supplementary Fig. 10 PFOS-induced AIM2 inflammasome activation is independent of mtROS generation. a-f THP-1-derived macrophages (**a**) or Bone marrow-derived macrophages (BMDMs) (**b**) were treated with PFOS (150 μM, 6 h) or primed with LPS (200 ng/ml, 3 h) followed by ATP (5 mM, 6 h), the mitochondrial ROS generation was assessed by flow cytometry of cells stained with MitoSOX. ELISA analysis of IL-1β secretion from supernatants of THP-1-derived macrophages (**c**) or Bone marrow-derived

macrophages (BMDMs) (**d**) and treated with PFOS, LPS plus ATP, PFOS plus Mito-TEMPO (500 μM/1 h; THP-1: red plots; BMDMs: blue polts) or LPS plus ATP plus Mito-TEMPO. The maturation of IL-1β (p17) in the cell supernatants and pro-IL-1β in the cell lysates of THP-1-derived macrophages (**e**) or Bone marrow-derived macrophages (BMDMs) (**f**) as indicated were determined by immunoblot. In (**a-d**), all error bars, mean values ± SEM, P-values were determined by unpaired two-tailed Student's t test of n=3 independent biological experiment. For (**e** and **f**), similar results are obtained for three independent biological experiments. Source data are provided as a Source Data file.

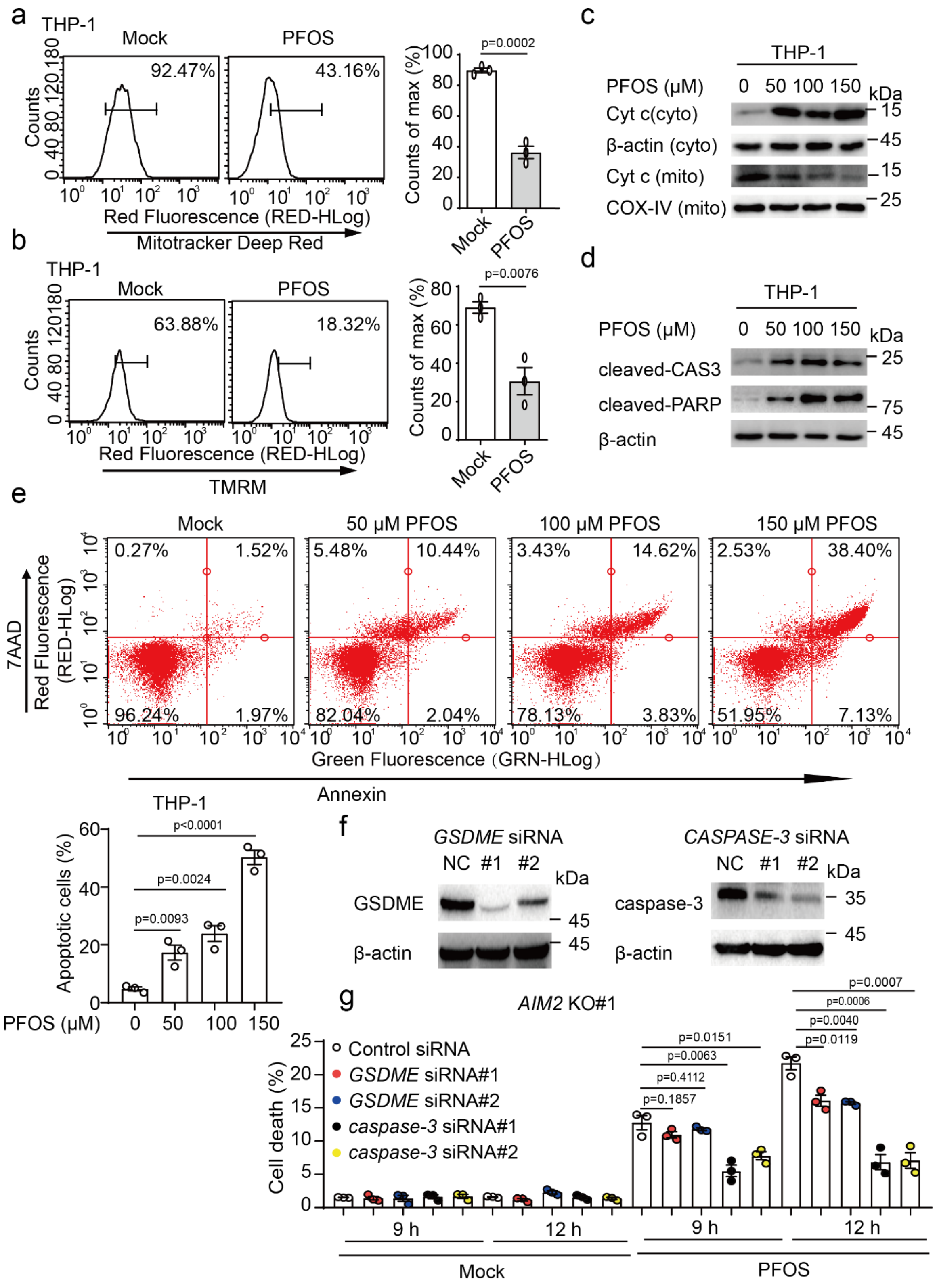

Supplementary Fig. 11 PFOS triggers mitochondrial dysfunction and apoptosis in THP-1-derived macrophages. a-b THP-1-derived macrophages were treated with PFOS

(150 μM, 6 h), and the mitochondrial respiration and membrane potential were assessed by flow cytometry of cells stained with Mitotracker deep red (**a**) and tetramethylrhodamine methyl ester (TMRM) (**b**), respectively. **c-d** Immunoblot analysis of Cytochrome c (Cyt c) from cytosolic extracts and mitochondria (**c**) or cleaved-capase-3 (cleaved-CAS3) and cleaved- PARP (**d**) derived from THP-1-derived macrophages stimulated with PFOS. **e** Apoptosis was assayed with Annexin V/7AAD staining of THP-1-derived macrophages with indicated dose of PFOS for 6 h. **f-g** *GSDME* knockdown (*GSDME* siRNA#1: red plots; #2: blue plots) or *CASPASE-3* knockdown (*CASPASE-3* siRNA#1: black plots; #2: yellow plots) in *AIM2* knockout (KO)-THP-1-derived macrophages were treated with PFOS (150 μM, 6 h) as indicated time. Cell lysates were collected to determine the knockdown efficiency of *GSDME* or *CASPASE-3* by immnoblot (**f**). Culture supernatant was then harvested to measure cell death by LDH release (**g**). In (**a**, **b**, **e** and **g**), all error bars, mean values ± SEM, P-values were determined by unpaired two-tailed Student's t test of n=3 independent biological experiment. For (**c**-**d** and **f**), similar results are obtained for three independent biological experiments. Source data are provided as a Source Data file.

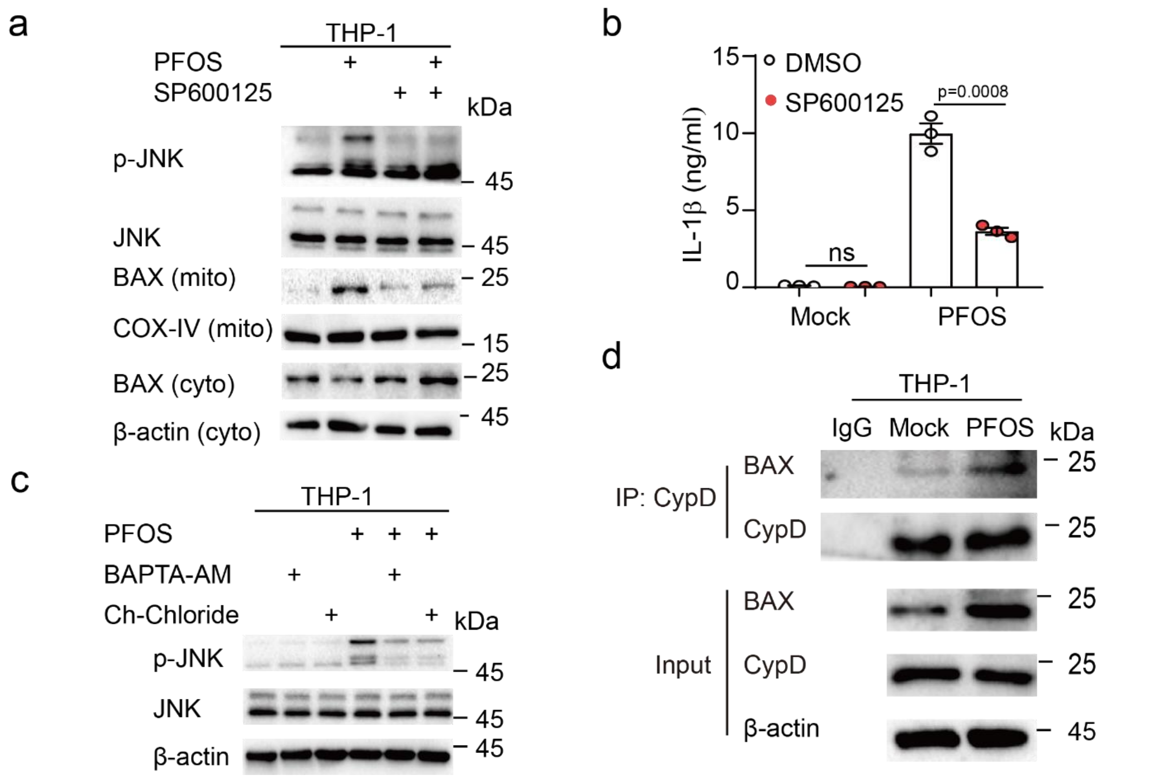

Supplementary Fig. 12 PFOS triggers Ca^{2+}-PKC-JNK pathway-dependent BAX activation and induces the interaction between BAX-CypD. a-b THP-1-derived macrophages were pretreated with JNK specific inhibitor SP600125 (40 μM for 1 h, red plots) and subsequently treated with PFOS (150 μM, 6 h). Cell lysates were collected to detect the phosphorylation of JNK and BAX tranlocation (**a**). Cell supernatants were collected to measure IL-1β production by ELISA (**b**). **c** THP-1-derived macrophages were pretreated with Ca^{2+} chelator BAPTA-AM (50 μM, 1 h) or PKC inhibitor chelerythrine chloride (Ch-Chloride, 10 μM, 1 h) and subsequently treated with PFOS (150 μM, 6 h). Cell lysates were collected for immunoblot analysis. **d** THP-1-derived macrophages were treated with PFOS (150 μM, 6 h) and then cell lysates were subjected to immunoprecipitation with anti-CypD and immunoblot with anti-BAX. For (**a** and **c-d**), similar results are obtained for three independent biological experiments. In **b**, error bars, mean values ± SEM, P-values were determined by unpaired two-tailed Student's t test of n=3 independent biological experiment. Source data are provided as a Source Data file.

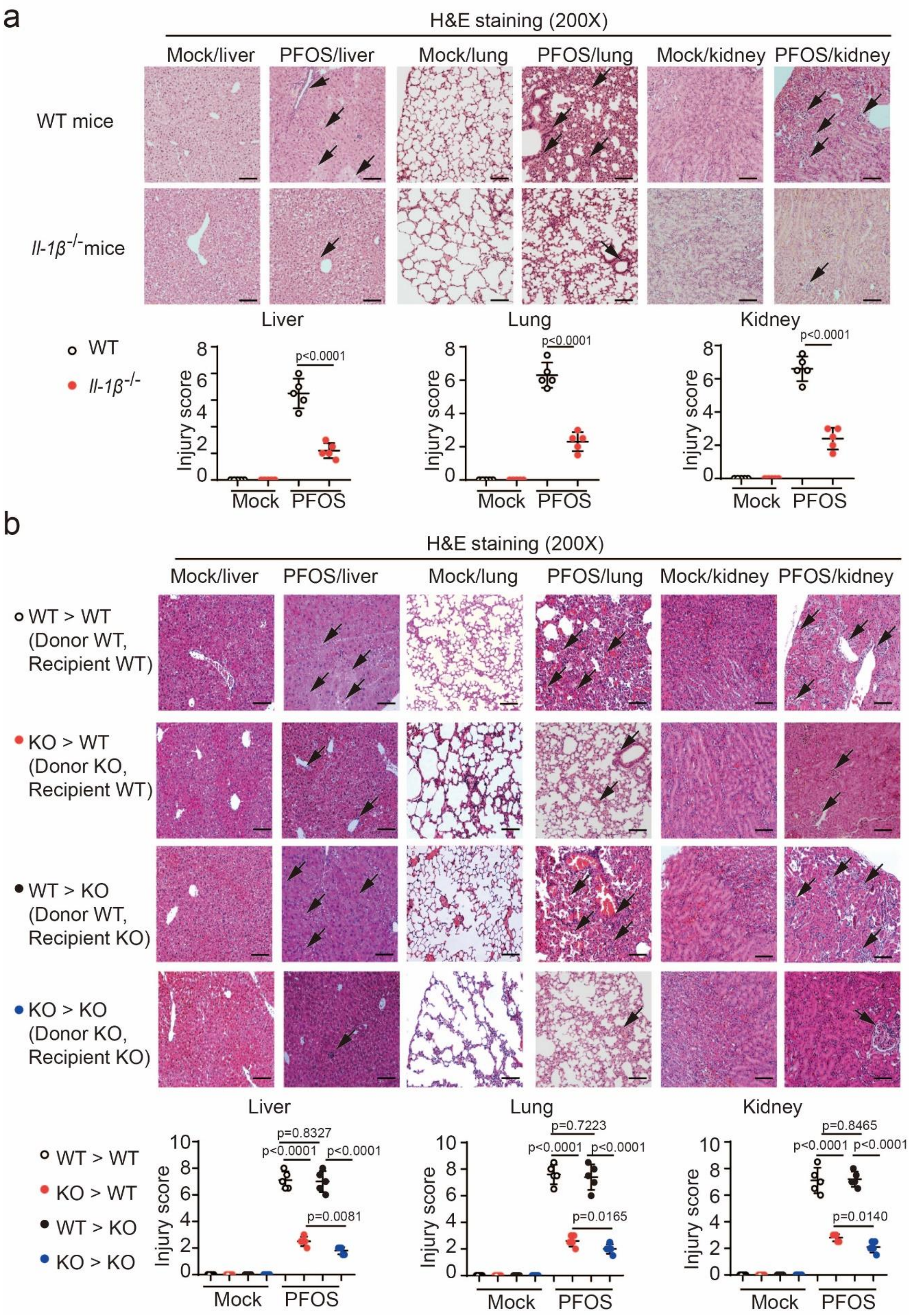
a
H&E staining (200X)
Mock/liver
PFOS/liver
Mock/lung
PFOS/lung
Mock/kidney
PFOS/kidney
WT mice
Il-1β-/- mice
Liver
Lung
Kidney
WT
Il-1β-/-
Injury score
p<0.0001
Mock
PFOS
b
H&E staining (200X)
WT > WT (Donor WT, Recipient WT)
KO > WT (Donor KO, Recipient WT)
WT > KO (Donor WT, Recipient KO)
KO > KO (Donor KO, Recipient KO)
p=0.8327
p=0.0081
p=0.7223
p=0.0165
p=0.8465
p=0.0140
WT > WT
KO > WT
WT > KO
KO > KO

Supplementary Fig. 13 The effects of IL-1β-dependent tissue injury by PFOS expsoure and the role of AIM2 in myeloid-derived cells on PFOS-induced inflammatory responses *in vivo*. a-b Wild type (WT) and *IL-1β*$^{-/-}$ (red plots) mice (female, 6 weeks old, n=5) (**a**) or the indicated bone marrow chimera mice (**b**, female, 6 weeks old, n=5) (KO > WT: red plots; WT > KO: black plots; KO > KO: blue plots) were i.p. with PBS containing 2 % Tween-80 (Mock) or PFOS (25 mg/kg body weight per day). At 5 days post-treatment, liver tissue, lung tissue and kidney tissue of these mice were stained with hematoxylin-eosin (H&E) and assayed using a light microscope with ×200 mangnification. Scale bar, 100 μm. The tissue (liver, lung, and kidney) injury score was determined and averaged in 5 randomly selected nonoverlapping fields from respective individual mouse tissue sections. All histology analyses were conducted in a blinded manner. All error bars, mean values ± SD, P-values were determined by unpaired two-tailed Student's t test of n=5 independent biological mice per group. Source data are provided as a Source Data file.

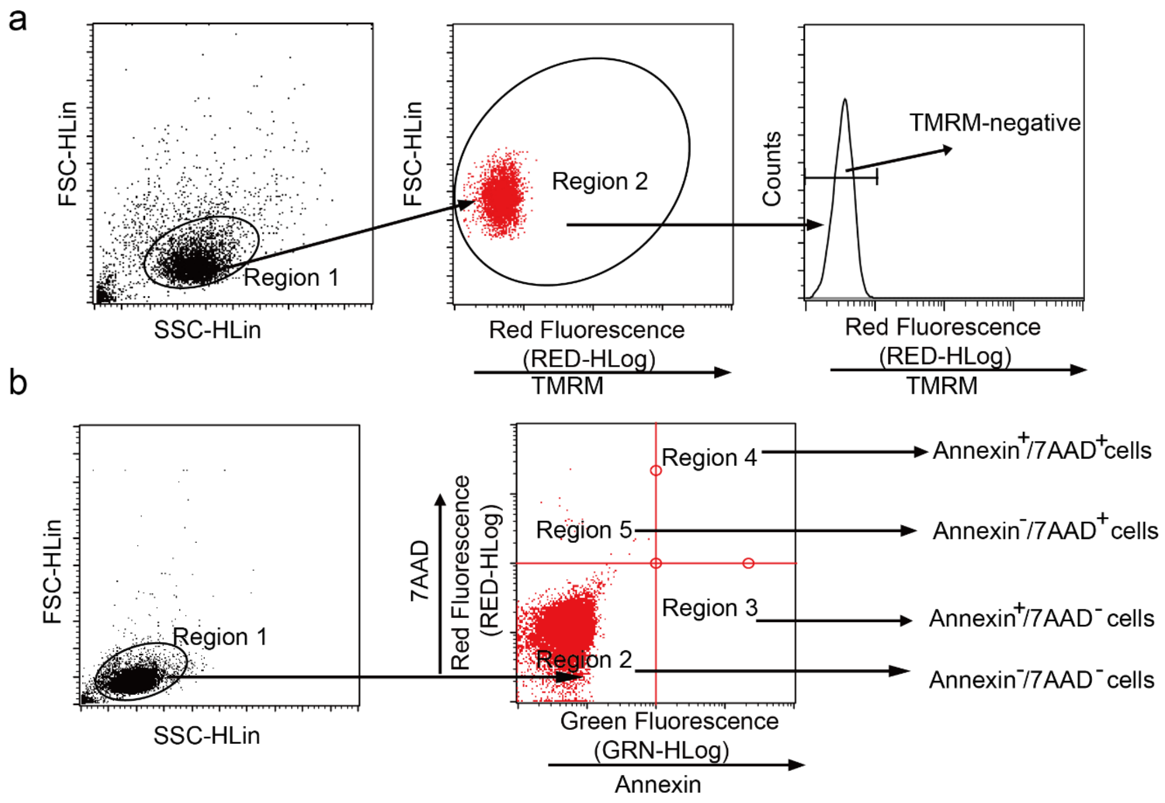

Supplementary Fig. 14 Gating strategies used for apoptotic cells and TMRM-negative/positive cells detection. (**a**) Gating strategy to assay TMRM-positive cells of Fig.4b and Supplementary Fig.11b: left, Region 1 indicating starting cells of non-staining sample were gated by FSC-HLin/SSC-HLin; middle: Region 2 indicating all cells from Region 1 were gated by FSC-HLin/ Red Fluorescence (RED-HLog); right: TMRM-negative cells. The same strategy was used to detect the Mitotracker Deep Red- positive cells presented in Fig.4a and Supplementary Fig.11a and MitoSox-positive cells presented in Supplementary Fig.10a and b. (**b**) Gating strategy to assay apoptotic cells: left, starting cells of non-staining samples were gated by FSC-HLin/SSC-HLin; right, all cells from Region 1 were gated by Red Fluorescence (RED-HLog)/ Green Fluorescence (GRN-HLog). The cells in the Region 2 are indicated as living cells and the living cells of the non-staining sample is > 98%. The cells in the Region 3, 4 and 5 are indicated as early apoptotic cells, intermediate/late apoptotic cells and necrotic cells, respectively. The total of apoptotic cells of sample (%) = cells (%) in the Region 3 + cells (%) in the Region 4.

Supplementary Table 1. Dilution of antibodies used in this study

Antibodies	Source	Identifier	Dilution
Anti-Flag (M2)	Sigma-Aldrich	Cat# F1804	1:3000 for WB
Anti-β-actin	Sigma-Aldrich	Cat# A1978	1:4000 for WB
Anti-Bip	Beyotime Technology	Cat# AB310	1:1000 for WB
Anti-AIM2	Abcam	Cat# ab93105	1:1000 for WB<br>1:400 for IP
Anti-COX-IV	Abcam	Cat# ab16056	1:1000 for WB
Anti-ASC	Santa Cruz Biotechnology	Cat# sc-271054	1:1000 for WB
Anti-caspase-1	Santa Cruz Biotechnology	Cat# sc-515	1:1000 for WB
Anti-Bcl-2	Santa Cruz Biotechnology	Cat# sc-7382	1:1000 for WB
Anti-GSDMD	Santa Cruz Biotechnology	Cat# sc-81868	1:1000 for WB
Anti-GSDME	Santa Cruz Biotechnology	Cat# sc-393162	1:1000 for WB
Anti-Cyclophilin D/ Cyclophilin F	Santa Cruz Biotechnology	Cat# sc-376061	1:1000 for WB<br>1:400 for IP
Goat anti-mouse	Santa Cruz Biotechnology	Cat# sc-2005	1:4000 for WB
Goat anti-rabbit	Santa Cruz Biotechnology	Cat# sc-2004	1:4000 for WB
Anti-JNK	Cell Signaling Technology	Cat# 9252	1:1000 for WB
Anti-phospho-SAPK/JNK	Cell Signaling Technology	Cat# 9255	1:1000 for WB
Anti-BAX	Cell Signaling Technology	Cat# 5023	1:1000 for WB<br>1:400 for IP

Anti-BAK	Cell Signaling Technology	Cat# 12105	1:1000 for WB
Anti-Cytochrome c	Cell Signaling Technology	Cat# 4272	1:1000 for WB
Anti-cleaved caspase-3	Cell Signaling Technology	Cat# 9664	1:1000 for WB
Anti-cleaved PARP	Cell Signaling Technology	Cat# 5625	1:1000 for WB
Anti-IL-1β	Cell Signaling Technology	Cat# 12242	1:1000 for WB
Anti-caspase-1	Cell Signaling Technology	Cat# 2225	1:1000 for WB
Anti-IκBα	Cell Signaling Technology	Cat# 4814	1:1000 for WB
Anti-p65	Cell Signaling Technology	Cat# 6956	1:1000 for WB
Anti-p-p65	Cell Signaling Technology	Cat# 3033	1:1000 for WB
Anti-NLRP3	AdipoGen	Cat# A27391510	1:3000 for WB<br>1:400 for IP

Supplementary Table 2. Sequences of PCR primers used in this study

Gene Name	Primer	Sequence
Human *IL-1β*	Forward	5'-AGCTACGAATCTCCGACCAC-3'
	Reverse	5'-CGTTATCCCATGTGTCGAAGAA-3'
Human *TNF-α*	Forward	5'-GAGGCCAAGCCCTGGTATG-3'
	Reverse	5'-CGGGCCGATTGATCTCAGC-3'
Human *IL-6*	Forward	5'-ACTCACCTCTTCAGAACGAATTG-3'
	Reverse	5'-CCATCTTTGGAAGGTTCAGGTTG-3'
Human *BCL-2*	Forward	5'-GGTGGGGTCATGTGTGTGG-3'
	Reverse	5'-CGGTTCAGGTACTCAGTCATCC-3'
Human *BAX*	Forward	5'-CCCGAGAGGTCTTTTTCCGAG-3'
	Reverse	5'-CCAGCCCATGATGGTTCTGAT-3'
Human *BAK*	Forward	5'-GTTTTCCGCAGCTACGTTTTT-3'
	Reverse	5'-GCAGAGGTAAGGTGACCATCTC-3'
Human *GAPDH*	Forward	5'-GGAGCGAGATCCCTCCAAAAT-3'
	Reverse	5'-GGCTGTTGTCATACTTCTCATGG-3'
Mouse *Il-1β*	Forward	5'-GCAACTGTTCCTGAACTCAACT-3'
	Reverse	5'-ATCTTTTGGGGTCCGTCAACT-3'
Mouse *Tnf-α*	Forward	5'-GACGTGGAACTGGCAGAAGAG-3'
	Reverse	5'-TTGGTGGTTTGTGAGTGTGAG-3'
Mouse *Il-6*	Forward	5'-TAGTCCTTCCTACCCCAATTTCC-3'
	Reverse	5'-TTGGTCCTTAGCCACTCCTTC-3'
Mouse *Gapdh*	Forward	5'-AGGTCGGTGTGAACGGATTTG-3'
	Reverse	5'-TGTAGACCATGTAGTTGAGGTCA-3'
Human *D-loop*	Forward	5'-GTTTATGTAGCTTACCTCCTC-3'
	Reverse	5'-TTGTTTATGGGGTGATGTGAG-3'
Human *Tert*	Forward	5'-TCACGGAGACCACGTTTCAAA-3'
	Reverse	5'-TTCAAGTGCTGTCTGATTCCAAT-3'
Mouse *D-loop*	Forward	5'-AATCTACCATCCTCCGTGAAACC-3'
	Reverse	5'-TCAGTTTAGCTACCCCCAAGTTTAA-3'

Mouse *Tert*	Forward	5'- CTAGCTCATGTGTCAAGACCCTCTT-3'
	Reverse	5’- GCCAGCACGTTTCTCTCGTT-3'
EGFP	Forward	5'-ACGGCGACGTAAACGGCCAC-3'
	Reverse	5'-GCACGCCGTAGGCTAGGGTG-3'
Human *mtDNA1*	Forward	5'-CACCCAAGAACAGGGTTTGT-3'
	Reverse	5'-TGGCCATGGGTATGTTGTTAA-3'
Human *mtDNA2*	Forward	5'-CTATCACCCTATTAACCACTCA-3'
	Reverse	5'-TTCGCCTGTAATATTGAACGTA-3'
Human *mtDNA3*	Forward	5'-AATCGAGTAGTACTCCCGATTG-3'
	Reverse	5'-TTCTAGGACGATGGGCATGAAA-3'
Human *Myc*	Forward	5'-AAGGACTATCCTGCTGCCAA-3
	Reverse	5'-CCTCTTGACATTCTCCTCGG-3'
Human *Sox2*	Forward	5'-TTTTGTCGGAGACGGAGAAG-3'
	Reverse	5'-CATGAGCGTCTTGGTTTTCC-3'
Human *18S*	Forward	5'- TAGAGGGACAAGTGGCGTTC-3'
	Reverse	5'- CGCTGAGCCAGTCAGTGT -3'
