# Supplementary material for: Perfluoroalkyl substance pollutants activate the innate immune system through the AIM2 inflammasome
Source: Nat Commun. 2021 May 18;12:2915. doi: 10.1038/s41467-021-23201-0 (PMC8131593; doi:10.1038/s41467-021-23201-0)
Supplement: Supplementary file 3 — Reporting Summary [file 41467_2021_23201_MOESM3_ESM.pdf]

nature research

Corresponding author(s): Jun Cui

Last updated by author(s): Feb 10, 2021

Reporting Summary

Nature Research wishes to improve the reproducibility of the work that we publish. This form provides structure for consistency and transparency in reporting. For further information on Nature Research policies, see our Editorial Policies and the Editorial Policy Checklist.

Statistics

For all statistical analyses, confirm that the following items are present in the figure legend, table legend, main text, or Methods section.

n/a	Confirmed	
☐	☒	The exact sample size (*n*) for each experimental group/condition, given as a discrete number and unit of measurement
☐	☒	A statement on whether measurements were taken from distinct samples or whether the same sample was measured repeatedly
☐	☒	The statistical test(s) used AND whether they are one- or two-sided<br>*Only common tests should be described solely by name; describe more complex techniques in the Methods section.*
☒	☐	A description of all covariates tested
☒	☐	A description of any assumptions or corrections, such as tests of normality and adjustment for multiple comparisons
☐	☒	A full description of the statistical parameters including central tendency (e.g. means) or other basic estimates (e.g. regression coefficient) AND variation (e.g. standard deviation) or associated estimates of uncertainty (e.g. confidence intervals)
☐	☒	For null hypothesis testing, the test statistic (e.g. *F*, *t*, *r*) with confidence intervals, effect sizes, degrees of freedom and *P* value noted<br>*Give P values as exact values whenever suitable.*
☒	☐	For Bayesian analysis, information on the choice of priors and Markov chain Monte Carlo settings
☒	☐	For hierarchical and complex designs, identification of the appropriate level for tests and full reporting of outcomes
☒	☐	Estimates of effect sizes (e.g. Cohen's *d*, Pearson's *r*), indicating how they were calculated

Our web collection on statistics for biologists contains articles on many of the points above.

Software and code

Policy information about availability of computer code

Data collection	H&E staining and PAS staining images were examined using a microscope (DMi8; Leica). For western blotting, ChemiDoc MP System was used for protein detection (BD Biosciences). Flow cytometry data were acquired using an guava easyCyte HT system (Millipore).
Data analysis	Image LAb software version 6.0 (Bio-Rad, Hercules,USA); ImageJ software version 1.52 (National Institutes of Health, Maryland,USA); GraphPad Prism software version 8.0 (GraphPad; California,USA); Flowjo software 10.0 (Tree Star, Ashland, USA)

For manuscripts utilizing custom algorithms or software that are central to the research but not yet described in published literature, software must be made available to editors and reviewers. We strongly encourage code deposition in a community repository (e.g. GitHub). See the Nature Research guidelines for submitting code & software for further information.

Data

Policy information about availability of data

All manuscripts must include a data availability statement. This statement should provide the following information, where applicable:

- Accession codes, unique identifiers, or web links for publicly available datasets
- A list of figures that have associated raw data
- A description of any restrictions on data availability

The authors declare that the data supporting the findings of this study are available within the paper and its supplementary information files.

Field-specific reporting

Please select the one below that is the best fit for your research. If you are not sure, read the appropriate sections before making your selection.

☒ Life sciences ☐ Behavioural & social sciences ☐ Ecological, evolutionary & environmental sciences

For a reference copy of the document with all sections, see nature.com/documents/nr-reporting-summary-flat.pdf

Life sciences study design

All studies must disclose on these points even when the disclosure is negative.

Sample size	Sample size for each experiment is indicated in the legend. No statistical methods were used to predetermine sample sizes. Sample size was chosen based on previous experiments and comparable. The reference of cellular experiments sample size is: Liu T, et al. TRIM11 Suppresses AIM2 Inflammasome by Degrading AIM2 via p62-Dependent Selective Autophagy. Cell Rep volume16, pages 1988-2002 (2016). The reference of in vivo experiments is: Dang EV, et al. Oxysterol Restraint of Cholesterol Synthesis Prevents AIM2 Inflammasome Activation. Cell volume 171, pages 1057-1071 (2017).
Data exclusions	No exclusion of data was made.
Replication	All experimental findings were reproduced in multiple independent experiments. For each figure, the number of independent experiments or biological replicates is indicated in the figure legends. Western blot and microscopy pictures are from a representative experiment and the number of independent repeats is clearly indicated in the figure legends.
Randomization	No statistical methods were used for randomization. For in vitro experiments, bone marrow-derived macrophages were isolated from randomly chosen 6 weeks old of wild-type or Nlrp3-/- or Aim2-/- mice. For in vivo experiments, 6 weeks old of wild-type or Nlrp3-/- or Aim2-/- mice were randomly allocated into experimental groups.
Blinding	Investigators were blinded to group allocation during data collection. Investigators were blinded for analysis of histological specimens. In experiments without subjective estimation like flow cytometry and qPCR, investigators were unblinded since no bias would be introduced by the investigators.

Reporting for specific materials, systems and methods

We require information from authors about some types of materials, experimental systems and methods used in many studies. Here, indicate whether each material, system or method listed is relevant to your study. If you are not sure if a list item applies to your research, read the appropriate section before selecting a response.

Materials & experimental systems

n/a	Involved in the study	
☐	☒	Antibodies
☐	☒	Eukaryotic cell lines
☒	☐	Palaeontology and archaeology
☐	☒	Animals and other organisms
☒	☐	Human research participants
☒	☐	Clinical data
☒	☐	Dual use research of concern

Methods

n/a	Involved in the study	
☒	☐	ChIP-seq
☐	☒	Flow cytometry
☒	☐	MRI-based neuroimaging

Antibodies

Antibodies used	Anti-Bip (Cat.AB310,1:1000) was from Beyotime Technology. Anti-AIM2 (Cat.ab93015,1:1000), and anti-COX-IV (Cat.ab16056,1:1000) was from Abcam. Anti-ASC (Cat.sc-271054,1:1000), anti-caspase-1p10 (Cat.sc-515,1:1000), anti-Bcl-2 (Cat.sc-7382,1:1000), anti-GSDMD (Cat.sc-81868,1:1000), anti-GSDME (Cat.sc-393162,1:1000), anti-Cyclophilin F (Cat.sc-376061,1:1000), goat anti-mouse (Cat.sc-2005,1:4000) and goat anti-rabbit (Cat.sc-2004,1:4000) were purchased from Santa Cruz Biotechnology. Anti-JNK (Cat.9252,1:1000), anti-p-SAPK/JNK (Cat.9255,1:1000), anti-BAX (Cat.5023,1:1000), anti-BAK (Cat.#12105,1:1000), anti-cyt C (Cat.#4272,1:1000), anti-IL-1β (Cat.#12242,1:1000) and anti-caspase-1 (Cat.#2225,1:1000), anti-caspase-3 (9662S,1:1000), anti-cleaved caspase-3 (Cat.#9664S,1:1000), anti-cleaved PARP (Cat.#5625S,1:1000), anti-IκBα (Cat.#4814,1:1000), anti-p65/RelA (Cat.#6956,1:1000) and anti-p-p65 (Cat.#3033,1:1000) were purchased from Cell Signaling Technology. Anti-NLRP3 (A27391510,1:2000) was purchased from AdipoGen. Anti-Flag (Cat. F1804,1:3000) and anti-β-actin (A1978, 1:4000) were purchased from Sigma-Aldrich.
Validation	All antibodies were obtained from indicated commercial vendors with ensured quality. All the antibodies used in this study have been validated by the vendors as indicated on the websites. Citations are listed as below: Anti-Bip (Beyotime Technology, cat. AB310) validate in human and mouse for WB: Yao W, et al. Endoplasmic reticulum stress links

hepatitis C virus RNA replication to wild-type PGC-1α/liver-specific PGC-1α upregulation. J Virol. 88(15):8361-74 (2014).
Anti-AIM2 (Abcam, cat. ab93015) validate in human and mouse for WB/IP:
Wang J, et al. HMGB1 participates in LPS-induced acute lung injury by activating the AIM2 inflammasome in macrophages and inducing polarization of M1 macrophages via TLR2, TLR4, and RAGE/NF-kB signaling pathways. Int J Mol Med 45:61-80 (2020).
Anti-COX-IV (Abcam, cat. ab16056) validate in human and mouse for WB:
Zhang XH et al. Heat shock protein 90 relieves heat stress damage of myocardial cells by regulating Akt and PKM2 signaling in vivo. Int J Mol Med 45:1888-1908 (2020).
Anti-ASC (Santa Cruz Biotechnology, cat. sc-271054) validate in human and mouse for WB:
Zhang L, et al. NLRP3 inflammasome inactivation driven by miR 223 3p reduces tumor growth and increases anticancer immunity in breast cancer. Mol Med Rep. 19(3):2180-2188 (2019). Choi YJ, et al. Publisher Correction: SERPINB1-mediated checkpoint of inflammatory caspase activation. Nat Immunol. 2019.
Anti-caspase-1 (Santa Cruz Biotechnology, cat. sc- 515) validate in human for WB: Liu T, et al. TRIM11 Suppresses AIM2 Inflammasome by Degrading AIM2 via p62-Dependent Selective Autophagy. Cell Rep. 16(7):1988-2002 (2016).
Anti-Bcl-2 (Santa Cruz Biotechnology, cat. sc-7382) validate in human and mouse for WB: Kong J, et al. Effect of microRNA-29b on proliferation, migration, and invasion of endometrial cancer cells. J Int Med Res. 47(8):3803-3817 (2019). Jin J, et al. LRG1 Promotes Apoptosis and Autophagy through the TGFβ-smad1/5 Signaling Pathway to Exacerbate Ischemia/Reperfusion Injury. Neuroscience. 413:123-134 (2019).
Anti-GSDMD (Santa Cruz Biotechnology, sc-81868) validate in human and mouse for WB: Xue Z, et al. miR-21 promotes NLRP3 inflammasome activation to mediate pyroptosis and endotoxic shock. Cell Death Dis. 10(6):461(2019).
Anti-GSDME (Santa Cruz Biotechnology, sc-393162) validate in mouse for WB:
Poh L, et al. AIM2 inflammasome mediates hallmark neuropathological alterations and cognitive impairment in a mouse model of vascular dementia. Mol Psychiatry. 2020.
Anti-Cyclophilin F (Santa Cruz Biotechnology, sc-37606) validate in human for WB: Zhang R, et al. Hirsutine induces mPTP-dependent apoptosis through ROCK1/PTEN/PI3K/GSK3β pathway in human lung cancer cells. Cell Death Dis. 2018 9(6):598 (2018).
Anti-β-actin (Sigma-Aldrich, A1978) validate in human and mouse for WB: Zheng Y, et al. Zika virus elicits inflammation to evade antiviral response by cleaving cGAS via NS1-caspase-1 axis. EMBO J. (2018).
Anti-BAK (Cell Signaling Technology, cat.12105) validate in human and mouse for WB:
Zhu ZC, et al. XPO1 inhibitor KPT-330 synergizes with Bcl-xL inhibitor to induce cancer cell apoptosis by perturbing rRNA processing and Mcl-1 protein synthesis. Cell Death Dis. 10(6):395 (2019). Giampazolias E, et al. Mitochondrial permeabilization engages NF-κB-dependent anti-tumour activity under caspase deficiency. Nat Cell Biol 19(9):1116-1129 (2017).
Anti-BAX (Cell Signaling Technology, cat,5023) validates in human and mouse for WB: Xia X, Huang C, Liao Y, Liu Y, He J, Guo Z, Jiang L, Wang X, Liu J, Huang H. Inhibition of USP14 enhances the sensitivity of breast cancer to enzalutamide. J Exp Clin Cancer Res. 38(1):220 (2019).
Anti-JNK (Cell Signaling Technology, cat.9252), anti-p-SAPK/JNK (Cell Signaling Technology, cat.9255) and anti-IκBα (Cell Signaling Technology, cat.4814) validate in human for WB: Feng Y, Duan T, Du Y, Jin S, Wang M, Cui J, Wang RF. LRRC25 Functions as an Inhibitor of NF-κB Signaling Pathway by Promoting p65/RelA for Autophagic Degradation. Sci Rep. 18;7(1):13448 (2017).
Anti-Flag (Sigma-Aldrich, cat. F1804) validates in human for WB:
Qin Y, Su Z, Wu Y, Wu C, Jin S, Xie W, Jiang W, Zhou R, Cui J. NLRP11 disrupts MAVS signalosome to inhibit type I interferon signaling and virus-induced apoptosis. EMBO Rep. 18(12):2160-2171 (2017).
Anti-cyt C (Cell Signaling Technology, Cat. #4272) validates in human and mouse for WB: Packer LM, et al. Bcl-2 inhibitors enhance FGFR inhibitor-induced mitochondrial-dependent cell death in FGFR2-mutant endometrial cancer. Mol Oncol. 13(4):738-756 (2019). W H, et al. Metformin Promotes the Survival of Random-Pattern Skin Flaps by Inducing Autophagy via the AMPK-mTOR-TFEB signaling pathway. Int J Biol Sci. 15(2):325-340 (2019).
Anti-cleaved caspase-3 (Cell Signaling Technology, cat, #9664) validates in human and mouse for WB: Barialai L, et al. AMPK activation protects astrocytes from hypoxia induced cell death. Int J Mol Med. 45(5):1385-1396 (2020).
Anti-cleaved PARP (Cell Signaling Technology, cat,5625) validates in human and mouse for WB: Wang S, et al. PRDX2 protects against oxidative stress induced by H. pylori and promotes resistance to cisplatin in gastric cancer. Redox Biol. 28:101319 (2020).
Anti-IL-1β (Cell Signaling Technology, cat,12242) validates in human and mouse for WB: Mehto S, et al. The Crohn's Disease Risk Factor IRGM Limits NLRP3 Inflammasome Activation by Impeding Its Assembly and by Mediating Its Selective Autophagy. Mol Cell. 2019 Feb 7;73(3):429-445.e7(2019). Bu J, et al. Acacetin protects against cerebral ischemia-reperfusion injury via the NLRP3 signaling pathway. Neural Regen Res. 14(4):605-612 (2019).
Anti-caspase-1 (Cell Signaling Technology, cat,2225) validates in human and mouse for WB: Yin XF, et al. NLRP3 in human glioma is correlated with increased WHO grade, and regulates cellular proliferation, apoptosis and metastasis via epithelial-mesenchymal transition and the PTEN/AKT signaling pathway. Int J Oncol. 253(3):973-986 (2018). Kim SY, et al. Pro-inflammatory hepatic macrophages generate ROS through NADPH oxidase 2 via endocytosis of monomeric TLR4-MD2 complex. Nat Commun. 21;8(1):2247 (2017).
Anti-IκBα (Cell Signaling Technology, cat,4814) validates in human and mouse for WB: Keuss MJ, et al. Unanchored tri-NEDD8 inhibits PARP-1 to protect from oxidative stress-induced cell death. EMBO J. 38(6):e100024 (2019). Xue Z, et al. miR-21 promotes NLRP3 inflammasome activation to mediate pyroptosis and endotoxic shock. Cell Death Dis. 10(6):461 (2019).
Anti-caspase-3 (Cell Signaling Technology, cat, #9662) validates in human and mouse for WB: Wang K, et al. Targeting alkaline ceramidase 3 alleviates the severity of nonalcoholic steatohepatitis by reducing oxidative stress. Cell Death Dis. 11(1):28 (2020). Wang Y, et al. Targeted overexpression of the long noncoding RNA ODSM can regulate osteoblast function in vitro and in vivo. Cell Death Dis. 11(2):133 （2020).
Anti-p65 (Cell Signaling Technology, cat, #6956) validates in human and mouse for WB: Zheng J, Fan R, Wu H, Yao H, Yan Y, Liu J, Ran L, Sun Z, Yi L, Dang L, Gan P, Zheng P, Yang T, Zhang Y, Tang T, Wang Y. Directed self-assembly of herbal small molecules into sustained release hydrogels for treating neural inflammation. Nat Commun. 10(1):1604 (2019).
Anti-p-p65 (Cell Signaling Technology, cat, #3033) validates in human and mouse for WB: Farabaugh KT, et al. PACT-mediated PKR activation acts as a hyperosmotic stress intensity sensor weakening osmoadaptation and enhancing inflammation. Elife. 9: e52241 (2020). Choi I, et al. Microglia clear neuron-released α-synuclein via selective autophagy and prevent neurodegeneration. Nat Commun. 211(1):1386 (2020).
Anti-NLRP3 (AdipoGen, cat. A27391510) validates in human and mouse for WB: Liu T, et al. USP19 suppresses inflammation and promotes M2-like macrophage polarization by manipulating NLRP3 function via autophagy. Cell Mol Immunol. 2020.

Eukaryotic cell lines

Policy information about cell lines

Cell line source(s)	THP-1 cells were purchased from cell Bank of the Chinese Academy of Sciences (Shanghai, China).
Authentication	THP-1 cells were authenticated for STR DNA profiling by cell Bank of the Chinese Academy of Sciences (Shanghai, China), no further authentication performed in the laboratory.
Mycoplasma contamination	These cell lines have been tested for mycoplasma contamination by MycoAler Mycoplasma Detection Kit (R&D Systems, cat.CUL001B) and the results of detection showed that cultured cells were not contaimated by mycoplasma.
Commonly misidentified lines (See ICLAC register)	There is no any commonly misidentified cell lines in this study.

Animals and other organisms

Policy information about studies involving animals; ARRIVE guidelines recommended for reporting animal research

Laboratory animals	C57BL/6 Aim2-/- mice were kindly provided by Dr. Bin Sun, Shanghai Institute of Biochemistry and Cell Biology. C57BL/6 Nlrp3-/- mice and C57BL/6 Il-1b-/- mice were kindly provided by Dr. Rongbin Zhou, University of Science and Technology of China. C57BL/6 wild type (WT,GDMLAC-O7) mice were purchased from Guangzhou Medical Laboratory Animal Center. Animals were kept and bred in a specific-pathogen free (SPF) environment with standard conditions of temperature (20-26 °C) and humidity (40-70 %) under a strict 12 h light cycle (lights on at 08:00 a.m. and off 08:00 p.m.) at Sun Yat-sen University, approved all the experimental protocols concerning the handing of mice. Mouse bone marrow macrophages were obtained from 6 weeks old female mice. For in vivo experiments, 6 weeks old females were used.
Wild animals	No any wild animals were observed during this investigation.
Field-collected samples	No any field-collected samples were collected during this investigation.
Ethics oversight	Animals were kept and bred in a specific-pathogen free (SPF) environment at Sun Yat-sen University, approved all the experimental protocols concerning the handing of mice. All animal experiments protocols were approved by the Animal Care Committee of the Sun Yat-sen University.

Note that full information on the approval of the study protocol must also be provided in the manuscript.

Flow Cytometry

Plots

Confirm that:

☒ The axis labels state the marker and fluorochrome used (e.g. CD4-FITC).

☒ The axis scales are clearly visible. Include numbers along axes only for bottom left plot of group (a 'group' is an analysis of identical markers).

☐ All plots are contour plots with outliers or pseudocolor plots.

☒ A numerical value for number of cells or percentage (with statistics) is provided.

Methodology

Sample preparation	Fig 4a,4b and Supplementary Fig 11a, 11b: BMDMs or THP-1 derived macrophages were treated with PFOS, then the cells were stained with itotracker deep red or tetramethylrhodamine methyl ester (TMRM) to detect the mitochondrial respiration and membrane potential by flow cytometry, respectively.<br>Supplementary Fig 10a, 10b: THP-1-derived macrophages or BMDMs were treated with PFOS (150 μM, 6 h) or primed with LPS (200 ng/ml, 3 h) followed by ATP (5 mM, 6 h), the mitochondrial ROS generation was assessed by flow cytometry of cells stained with MitoSOX.<br>Supplementary Fig 11e: Apoptosis was assayed with Annexin V/7AAD staining by flow cytometry analysis of THP-1 derived macrophages with indicated dose of PFOS for 6 h.
Instrument	Flow cytometry data were acquired using an guava easyCyte HT system (Millipore).
Software	All data were analyzed by Flowjo software 10.0 (Tree Star, Ashland, USA)
Cell population abundance	No post-sorting analysis was done.
Gating strategy	Starting cells were gated by FSC/SSC gates. Gates indicating boundaries between "positive" and "negative" are according to the non-staining samples.

☒ Tick this box to confirm that a figure exemplifying the gating strategy is provided in the Supplementary Information.
